# Data on roasted coffee with specific defects analyzed by infrared-photoacoustic spectroscopy and chemometrics

**DOI:** 10.1016/j.dib.2018.08.013

**Published:** 2018-08-09

**Authors:** Rafael Carlos Eloy Dias, Patrícia Valderrama, Paulo Henrique Março, Maria Brigida Dos Santos Scholz, Michael Edelmann, Chahan Yeretzian

**Affiliations:** a Zurich University of Applied Sciences (ZHAW), Institute of Chemistry and Biotechnology, Coffee Excellence Center, Einsiedlerstrasse 31, CH – 8820 Wädenswil, Switzerland; b Federal Technological University of Paraná State – UTFPR, Post-Graduation Program in Food Technology – PPGTA, Via Rosalina Maria dos Santos, 1233, Postal Code 271, 87301-899, Campo Mourão, Paraná, Brazil; c Instituto Agronômico do Paraná – IAPAR, Technical Scientific Board, Rod, Celso Garcia Cid, km 375, Postal Code 481, 86001-970 Londrina, Paraná, Brazil

## Abstract

This article contains data related to the research article entitled “Quantitative assessment of specific defects in roasted ground coffee via infrared-photoacoustic spectroscopy” (Dias et al., 2018) [1]. A method potentially able for assessing the quality of roasted ground coffees is described in the origin paper. Infrared spectroscopy and photoacoustic detection (FTIR-PAS) associated with multivariate calibration were used. The samples were obtained blending whole and healthy coffee beans (*C. arabica* and *C. canephora*) with specific blends of defects, named *selections*, which contain broken, sour, and black beans, skin, woods and healthy beans still not collected. In addition to a reduction in commercial value, the presence of defects compromises the sensory attributes of coffee. On the other hand, selections are commonly found in coffee crops and can be added intentionally to the product. Twenty-five selections were used to obtain a panel of 154 blends. The FTIR-PAS spectra of each sample generated the prediction model of Partial Least Squares Regression parameters, which are also presented here.

**Specifications Table**

**Table:** 

Subject area	Agricultural science
More specific subject area	Food chemistry
Type of data	Table, figure/graph
How data was acquired	Trained coffee selectors from Instituto Agronômico do Paraná – IAPAR – Brazil classified the selections (mixtures of healthy coffee beans and defects of coffee). Samples spectra were acquired using a Bruker FTIR spectrometer (Billerica, USA) - Tensor 37, coupled to a Gasera photoacoustic detector (Turku, Finland) - PA 301, interfaced with a DSP Module. Data were processed with MATLAB R2007 / PLS Toolbox 5.2 from Eigenvector Research.
Data format	Raw and analyzed.
Experimental factors	The raw coffee material were supplied by IAPAR. Samples of healthy beans of *Coffea arabica* (Arabica) and *Coffea canephora* (Robusta), namely *bases*, in three different proportions (0%, 20% and 50% of Robusta), and 25 blends of defective and healthy beans of Arabica, namely *selections*, were explored. Then, samples were obtained blending the bases with two levels of each selection (20% and 40%).
Experimental features	One hundred and fifty-four FTIR-PAS spectra (each spectrum is an average of three replicates, numbering 462 acquisitions) and their intensity values at 1763 wavelength values were organized as a 154 ×1763 matrix. PCA (Principal Component Analysis) and PLS-DA (Partial Least Squares with Discriminant Analysis) were then performed.
Data source location	The healthy coffee beans and selections were harvested/collected in Londrina – Paraná – Brazil: Latitude -23.29, Longitude -51.17; 23° 17′ 34′′ S, 51° 10׳ 24′′ W, humid subtropical climate.
Data accessibility	With this article
Related research article	Dias et al. [1]

**Value of the data**

• Data would be available as reference to agricultural and food science, notably for coffee quality researches.
• Representative coffee material based on real situations of harvesting were explored. These *selections* composed the samples also described herein. We are not aware of the occurrence of such information in the literature.
• The presented chemometric results are an interesting material for studies on multivariate statistical analysis.
• Data on chemometrics applied for FTIR-PAS spectra of roasted ground coffee with several levels of quality can be useful for future studies of coffee quality monitoring. Thus, this investigation is particularly interesting to regulatory and supervisory agencies.

## 1 Data

**Fig. 1:**
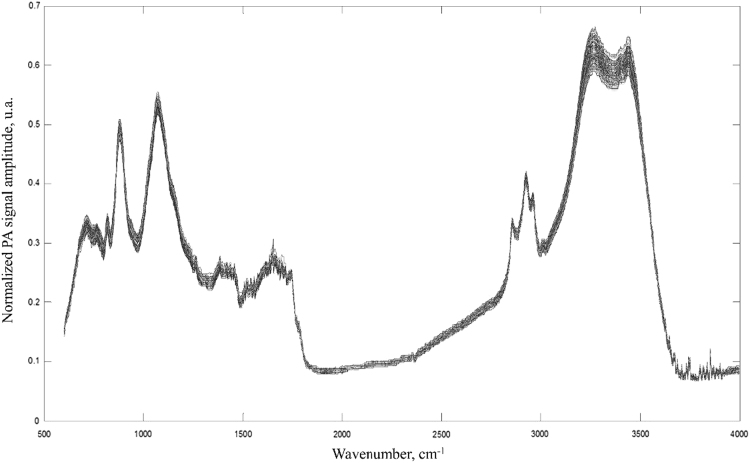
FTIR-PAS spectra of 154 samples of coffee (the sample set is provided in Table 2, and data spectra is given in Supplementary material).

**Fig. 2:**
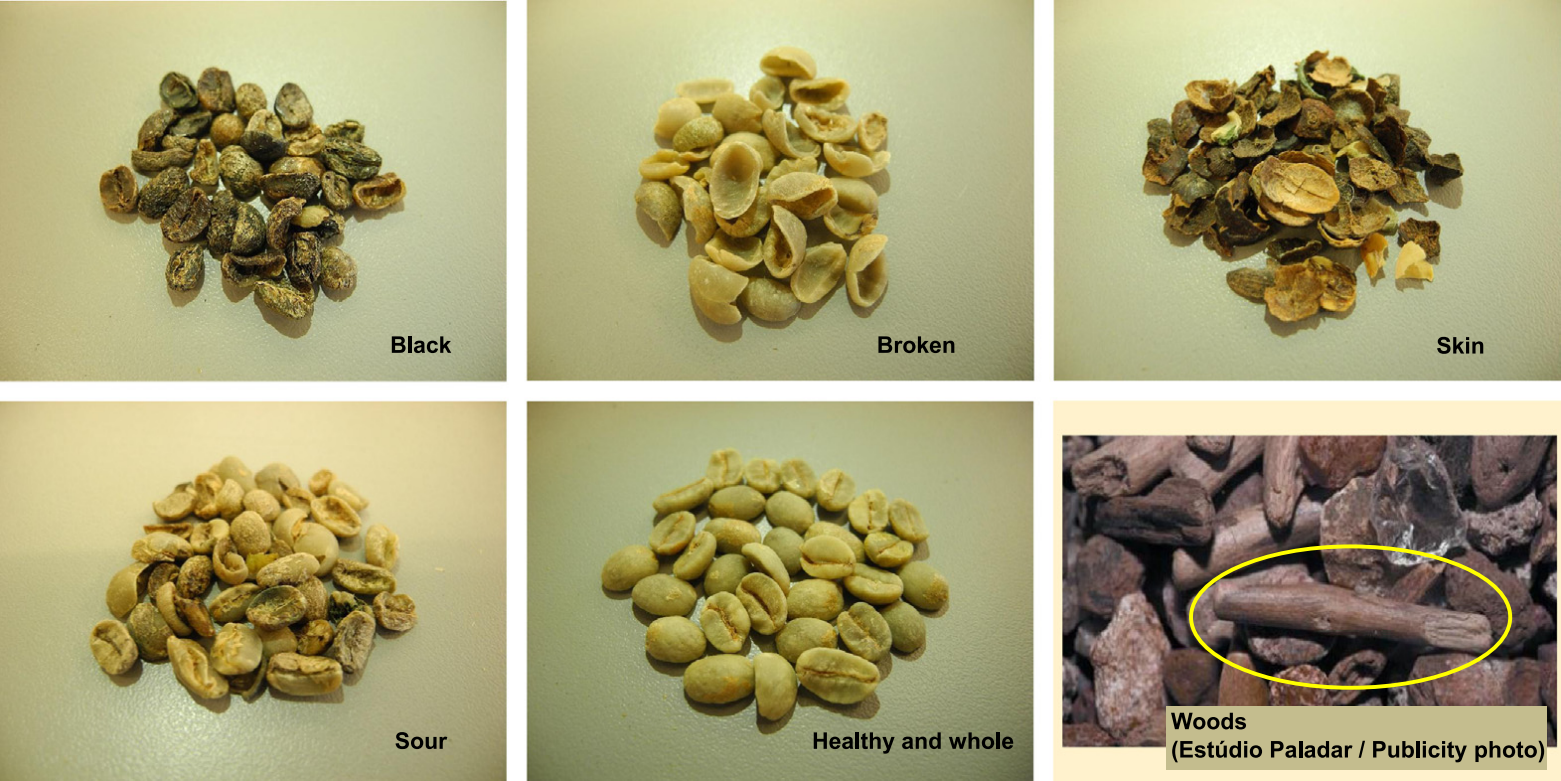
Photographs/images of defects of coffee. Woods picture is a publicity photo.

Table 1, Table 2 detail the selections composition and the composition of the samples obtained after blending healthy coffees and the selections in different proportions. Fig. 1 presents the FTIR-PAS spectra of 154 samples featured in Table 2. Table 3 brings PLS-DA model parameters. Images of coffee defects are found in Fig. 2. The spectra data of all samples are given as supplementary material.

**Table 1:** Composition of the coffee selections used to obtain the blends, in percentage (w/w).

Selection	Healthy^a^	Broken	Sour	Black	Skin^b^	Woods
1	10.58	37.40	37.43	13.23	1.35	0.10
2	3.65	11.78	31.64	43.04	9.93	0.08
3	19.18	17.40	47.25	11.04	5.40	0.00
4	19.70	19.60	55.13	5.14	1.00	0.00
5	14.97	21.87	48.51	13.54	1.19	0.19
6	57.14	2.63	31.92	8.42	0.00	0.00
7	7.11	16.23	55.89	19.16	1.85	0.46
8	13.65	6.82	51.19	27.44	0.65	0.05
9	7.27	14.41	45.06	32.82	0.26	0.00
10	11.09	28.17	53.54	6.03	0.71	0.00
11	4.62	0.64	51.12	42.66	0.00	0.00
12	7.57	10.49	66.68	9.08	5.39	0.50
13	9.43	16.28	53.18	10.16	10.78	0.00
14	2.71	19.78	67.97	8.30	0.97	0.00
15	0.60	7.64	73.54	15.33	1.71	0.25
16	3.09	19.09	50.15	7.68	19.60	0.20
17	1.41	8.78	51.71	22.41	15.70	0.05
18	21.21	16.01	52.89	5.30	3.74	0.40
19	10.48	17.51	52.37	16.31	2.52	0.05
20	4.16	16.97	59.66	15.29	4.14	0.20
21	12.32	12.99	49.70	5.65	19.08	0.44
22	0.60	9.65	43.29	37.84	9.09	0.48
23	17.63	13.25	57.89	10.43	0.58	0.19
24	4.22	9.39	61.29	20.50	3.84	0.86
25	7.21	7.69	58.93	14.35	6.69	5.10

a Healthy and whole beans (i.e. not broken).

b Also named seed coat, peel or exocarp.

**Table 2:** Final composition of the actual roasted ground coffee blends as evaluated by FTIR-PAS, after combining the selections with the bases. Included in the sample list are also the three pure bases (Basis A100, Basis AR20 and Basis AR50) and a sample of healthy Robusta coffee (R100).

Identification			Total whole^b^ and healthy beans (%)		Coffee defects (%)				
Sample^a^		Basis	Arabica	Robusta	Broken	Sour	Black	Skin	Woods
1	20A_1	A100%	82.1	0	7.49	7.49	2.65	0.27	0.02
2	20A_2	A100%	80.7	0	2.36	6.33	8.61	1.99	0.02
3	20A_3	A100%	83.8	0	3.48	9.45	2.21	1.08	0.00
4	20A_4	A100%	83.9	0	3.92	11.0	1.03	0.20	0.00
5	20A_5	A100%	83.0	0	4.38	9.72	2.71	0.24	0.04
6	20A_6	A100%	91.4	0	0.53	6.38	1.68	0.00	0.00
7	20A_7	A100%	81.4	0	3.26	11.2	3.85	0.37	0.09
8	20A_8	A100%	82.7	0	1.36	10.2	5.49	0.13	0.01
9	20A_9	A100%	81.4	0	2.88	9.01	6.56	0.05	0.00
10	20A_10	A100%	82.2	0	5.63	10.7	1.21	0.14	0.00
11	20A_11	A100%	80.9	0	0.13	10.2	8.53	0.00	0.00
12	20A_12	A100%	81.5	0	2.11	13.4	1.83	1.08	0.10
13	20A_13	A100%	81.8	0	3.26	10.7	2.03	2.16	0.00
14	20A_14	A100%	80.5	0	3.96	13.6	1.66	0.19	0.00
15	20A_15	A100%	80.1	0	1.53	14.7	3.07	0.34	0.05
16	20A_16	A100%	80.6	0	3.83	10.1	1.54	3.93	0.04
17	20A_17	A100%	80.2	0	1.76	10.4	4.48	3.14	0.01
18	20A_18	A100%	84.2	0	3.21	10.6	1.06	0.75	0.08
19	20A_19	A100%	82.1	0	3.50	10.5	3.26	0.50	0.01
20	20A_20	A100%	80.8	0	3.40	11.9	3.06	0.83	0.04
21	20A_21	A100%	82.4	0	2.61	10.0	1.13	3.83	0.09
22	20A_22	A100%	80.1	0	1.94	8.70	7.60	1.83	0.10
23	20A_23	A100%	83.5	0	2.66	11.6	2.09	0.12	0.04
24	20A_24	A100%	80.8	0	1.89	12.4	4.14	0.77	0.17
25	20A_25	A100%	81.5	0	1.62	12.4	3.02	1.41	1.02
26	40A_1	A100%	64.2	0	15.0	15.0	5.30	0.54	0.04
27	40A_2	A100%	61.4	0	4.72	12.7	17.2	3.98	0.03
28	40A_3	A100%	67.6	0	6.96	18.9	4.42	2.16	0.00
29	40A_4	A100%	67.8	0	7.84	22.1	2.06	0.40	0.00
30	40A_5	A100%	66.0	0	8.76	19.4	5.43	0.48	0.08
31	40A_6	A100%	82.9	0	1.05	12.8	3.37	0.00	0.00
32	40A_7	A100%	62.8	0	6.52	22.5	7.70	0.74	0.18
33	40A_8	A100%	65.4	0	2.73	20.5	11.0	0.26	0.02
34	40A_9	A100%	62.9	0	5.76	18.0	13.1	0.10	0.00
35	40A_10	A100%	64.4	0	11.3	21.4	2.41	0.28	0.00
36	40A_11	A100%	61.8	0	0.26	20.5	17.1	0.00	0.00
37	40A_12	A100%	63.0	0	4.22	26.8	3.65	2.17	0.20
38	40A_13	A100%	63.7	0	6.51	21.3	4.06	4.31	0.00
39	40A_14	A100%	61.0	0	7.91	27.3	3.32	0.39	0.00
40	40A_15	A100%	60.2	0	3.06	29.5	6.15	0.68	0.10
41	40A_16	A100%	61.2	0	7.65	20.1	3.08	7.86	0.08
42	40A_17	A100%	60.5	0	3.51	20.7	8.97	6.28	0.02
43	40A_18	A100%	68.5	0	6.43	21.2	2.13	1.50	0.16
44	40A_19	A100%	64.2	0	7.01	21.0	6.53	1.01	0.02
45	40A_20	A100%	61.6	0	6.80	23.9	6.13	1.66	0.08
46	40A_21	A100%	64.9	0	5.22	20.0	2.27	7.66	0.18
47	40A_22	A100%	60.2	0	3.88	17.4	15.2	3.65	0.19
48	40A_23	A100%	67.0	0	5.31	23.2	4.18	0.23	0.08
49	40A_24	A100%	61.7	0	3.79	24.7	8.27	1.55	0.34
50	40A_25	A100%	63.0	0	3.24	24.8	6.05	2.82	2.04
51	20AR20_1	A:R 80:20	66.1	16	7.49	7.49	2.65	0.27	0.02
52	20AR20_2	A:R 80:20	64.7	16	2.36	6.33	8.61	1.99	0.02
53	20AR20_3	A:R 80:20	67.8	16	3.48	9.45	2.21	1.08	0.00
54	20AR20_4	A:R 80:20	67.9	16	3.92	11.0	1.03	0.20	0.00
55	20AR20_5	A:R 80:20	67.0	16	4.38	9.72	2.71	0.24	0.04
56	20AR20_6	A:R 80:20	75.4	16	0.53	6.38	1.68	0.00	0.00
57	20AR20_7	A:R 80:20	65.4	16	3.26	11.2	3.85	0.37	0.09
58	20AR20_8	A:R 80:20	66.7	16	1.36	10.2	5.49	0.13	0.01
59	20AR20_9	A:R 80:20	65.4	16	2.88	9.01	6.56	0.05	0.00
60	20AR20_10	A:R 80:20	66.2	16	5.63	10.7	1.21	0.14	0.00
61	20AR20_11	A:R 80:20	64.9	16	0.13	10.2	8.53	0.00	0.00
62	20AR20_12	A:R 80:20	65.5	16	2.11	13.4	1.83	1.08	0.10
63	20AR20_13	A:R 80:20	65.8	16	3.26	10.6	2.03	2.16	0.00
64	20AR20_14	A:R 80:20	64.5	16	3.96	13.6	1.66	0.19	0.00
65	20AR20_15	A:R 80:20	64.1	16	1.53	14.7	3.07	0.34	0.05
66	20AR20_16	A:R 80:20	64.6	16	3.83	10.1	1.54	3.93	0.04
67	20AR20_17	A:R 80:20	64.2	16	1.76	10.4	4.48	3.14	0.01
68	20AR20_18	A:R 80:20	68.2	16	3.21	10.6	1.06	0.75	0.08
69	20AR20_19	A:R 80:20	66.1	16	3.50	10.5	3.26	0.50	0.01
70	20AR20_20	A:R 80:20	64.8	16	3.40	12.0	3.06	0.83	0.04
71	20AR20_21	A:R 80:20	66.4	16	2.61	10.0	1.13	3.83	0.09
72	20AR20_22	A:R 80:20	64.1	16	1.94	8.70	7.60	1.83	0.10
73	20AR20_23	A:R 80:20	67.5	16	2.66	11.6	2.09	0.12	0.04
74	20AR20_24	A:R 80:20	64.8	16	1.89	12.4	4.14	0.77	0.17
75	20AR20_25	A:R 80:20	65.4	16	1.53	12.3	2.92	1.33	1.02
76	40AR20_1	A:R 80:20	52.2	12	15.0	15.0	5.30	0.54	0.04
77	40AR20_2	A:R 80:20	49.4	12	4.72	12.7	17.2	3.98	0.03
78	40AR20_3	A:R 80:20	55.6	12	6.96	18.9	4.42	2.16	0.00
79	40AR20_4	A:R 80:20	55.8	12	7.84	22.1	2.06	0.40	0.00
80	40AR20_5	A:R 80:20	54.0	12	8.76	19.4	5.43	0.48	0.08
81	40AR20_6	A:R 80:20	70.9	12	1.05	12.8	3.37	0.00	0.00
82	40AR20_7	A:R 80:20	50.8	12	6.52	22.5	7.70	0.74	0.18
83	40AR20_8	A:R 80:20	53.4	12	2.73	20.5	11.0	0.26	0.02
84	40AR20_9	A:R 80:20	50.9	12	5.76	18.0	13.1	0.10	0.00
85	40AR20_10	A:R 80:20	52.4	12	11.3	21.4	2.41	0.28	0.00
86	40AR20_11	A:R 80:20	49.8	12	0.26	20.5	17.1	0.00	0.00
87	40AR20_12	A:R 80:20	51.0	12	4.22	26.8	3.65	2.17	0.20
88	40AR20_13	A:R 80:20	51.7	12	6.51	21.3	4.06	4.31	0.00
89	40AR20_14	A:R 80:20	49.0	12	7.91	27.2	3.32	0.39	0.00
90	40AR20_15	A:R 80:20	48.2	12	3.06	29.5	6.15	0.68	0.10
91	40AR20_16	A:R 80:20	49.2	12	7.65	20.1	3.08	7.86	0.08
92	40AR20_17	A:R 80:20	48.5	12	3.51	20.7	8.97	6.28	0.02
93	40AR20_18	A:R 80:20	56.5	12	6.43	21.2	2.13	1.50	0.16
94	40AR20_19	A:R 80:20	52.2	12	7.01	21.0	6.53	1.01	0.02
95	40AR20_20	A:R 80:20	49.6	12	6.80	23.9	6.13	1.66	0.08
96	40AR20_21	A:R 80:20	52.9	12	5.22	20.0	2.27	7.66	0.18
97	40AR20_22	A:R 80:20	48.2	12	3.88	17.4	15.2	3.65	0.19
98	40AR20_23	A:R 80:20	55.0	12	5.31	23.2	4.18	0.23	0.08
99	40AR20_24	A:R 80:20	49.7	12	3.79	24.7	8.27	1.55	0.34
100	40AR20_25	A:R 80:20	51.0	12	3.05	24.6	5.83	2.67	2.04
101	20AR50_1	A:R 50:50	42.1	40	7.49	7.49	2.65	0.27	0.02
102	20AR50_2	A:R 50:50	40.7	40	2.36	6.33	8.61	1.99	0.02
103	20AR50_3	A:R 50:50	43.8	40	3.48	9.45	2.21	1.08	0.00
104	20AR50_4	A:R 50:50	43.9	40	3.92	11.0	1.03	0.20	0.00
105	20AR50_5	A:R 50:50	43.0	40	4.38	9.72	2.71	0.24	0.04
106	20AR50_6	A:R 50:50	51.4	40	0.53	6.38	1.68	0.00	0.00
107	20AR50_7	A:R 50:50	41.4	40	3.26	11.2	3.85	0.37	0.09
108	20AR50_8	A:R 50:50	42.7	40	1.36	10.2	5.49	0.13	0.01
109	20AR50_9	A:R 50:50	41.4	40	2.88	9.01	6.56	0.05	0.00
110	20AR50_10	A:R 50:50	42.2	40	5.63	10.7	1.21	0.14	0.00
111	20AR50_11	A:R 50:50	40.9	40	0.13	10.2	8.53	0.00	0.00
112	20AR50_12	A:R 50:50	41.5	40	2.11	13.4	1.83	1.08	0.10
113	20AR50_13	A:R 50:50	41.8	40	3.26	10.6	2.03	2.16	0.00
114	20AR50_14	A:R 50:50	40.5	40	3.96	13.6	1.66	0.19	0.00
115	20AR50_15	A:R 50:50	40.1	40	1.53	14.7	3.07	0.34	0.05
116	20AR50_16	A:R 50:50	40.6	40	3.83	10.0	1.54	3.93	0.04
117	20AR50_17	A:R 50:50	40.2	40	1.76	10.4	4.48	3.14	0.01
118	20AR50_18	A:R 50:50	44.2	40	3.21	10.6	1.06	0.75	0.08
119	20AR50_19	A:R 50:50	42.1	40	3.50	10.5	3.26	0.50	0.01
120	20AR50_20	A:R 50:50	40.8	40	3.40	12.0	3.06	0.83	0.04
121	20AR50_21	A:R 50:50	42.4	40	2.61	10.0	1.13	3.83	0.09
122	20AR50_22	A:R 50:50	40.1	40	1.94	8.70	7.60	1.83	0.10
123	20AR50_23	A:R 50:50	43.5	40	2.66	11.6	2.09	0.12	0.04
124	20AR50_24	A:R 50:50	40.8	40	1.89	12.4	4.14	0.77	0.17
125	20AR50_25	A:R 50:50	41.4	40	1.53	12.3	2.92	1.33	1.02
126	40AR50_1	A:R 50:50	34.2	30	15.0	15.0	5.30	0.54	0.04
127	40AR50_2	A:R 50:50	31.4	30	4.72	12.7	17.2	3.98	0.03
128	40AR50_3	A:R 50:50	37.6	30	6.96	18.9	4.42	2.16	0.00
129	40AR50_4	A:R 50:50	37.8	30	7.84	22.1	2.06	0.40	0.00
130	40AR50_5	A:R 50:50	36.0	30	8.76	19.4	5.43	0.48	0.08
131	40AR50_6	A:R 50:50	52.9	30	1.05	12.8	3.37	0.00	0.00
132	40AR50_7	A:R 50:50	32.8	30	6.52	22.5	7.70	0.74	0.18
133	40AR50_8	A:R 50:50	35.4	30	2.73	20.5	11.0	0.26	0.02
134	40AR50_9	A:R 50:50	32.9	30	5.76	18.0	13.1	0.10	0.00
135	40AR50_10	A:R 50:50	34.4	30	11.3	21.4	2.41	0.28	0.00
136	40AR50_11	A:R 50:50	31.8	30	0.26	20.5	17.1	0.00	0.00
137	40AR50_12	A:R 50:50	33.0	30	4.22	26.8	3.65	2.17	0.20
138	40AR50_13	A:R 50:50	33.7	30	6.51	21.3	4.06	4.31	0.00
139	40AR50_14	A:R 50:50	31.0	30	7.91	27.2	3.32	0.39	0.00
140	40AR50_15	A:R 50:50	30.2	30	3.06	29.5	6.15	0.68	0.10
141	40AR50_16	A:R 50:50	31.2	30	7.65	20.1	3.08	7.86	0.08
142	40AR50_17	A:R 50:50	30.5	30	3.51	20.7	8.97	6.28	0.02
143	40AR50_18	A:R 50:50	38.5	30	6.43	21.3	2.13	1.50	0.16
144	40AR50_19	A:R 50:50	34.2	30	7.01	21.0	6.53	1.01	0.02
145	40AR50_20	A:R 50:50	31.6	30	6.80	23.9	6.13	1.66	0.08
146	40AR50_21	A:R 50:50	34.9	30	5.22	20.0	2.27	7.66	0.18
147	40AR50_22	A:R 50:50	30.2	30	3.88	17.4	15.2	3.65	0.19
148	40AR50_23	A:R 50:50	37.0	30	5.31	23.2	4.18	0.23	0.08
149	40AR50_24	A:R 50:50	31.7	30	3.79	24.7	8.27	1.55	0.34
150	40AR50_25	A:R 50:50	33.0	30	3.05	24.6	5.83	2.67	2.04
151	Basis A100	A100%	100	0	0	0	0	0	0
152	Basis AR20	A:R 80:20	80	20	0	0	0	0	0
153	Basis AR50	A:R 50:50	50	50	0	0	0	0	0
154	R100	R100%	0	100	0	0	0	0	0

a Samples identification: numbers 20 and 40 are the proportion of the selection (20% or 40%); the following letter(s) and numeral are the identification of the sample basis (Arabica coffee, A; Arabica/Robusta 80:20 w/w blend, AR20, or 50:50, AR50), and the last numeral is the selection identification (#1 – 25, from Table 1). For example, 20AR50_1 is the sample containing 20% of the selection #1 in the Arabica/Robusta 50:50 basis.

b The sum of whole and healthy beans from basis and of whole and healthy already included in selection (%).

**Table 3:** Root Mean Square Errors, sensitivity and specificity for the classes of PLS-DA model.

Classes	20A	40A	20AR20	40AR20	20AR50	40AR50
RMSEC	0.16059	0.22069	0.18578	0.20135	0.23597	0.15687
RMSEP	0.18120	0.21649	0.16222	0.22817	0.22455	0.15016
RMSECV	0.17965	0.24442	0.20895	0.22635	0.25309	0.16736
Sensitivity	1.000	1.000	1.000	0.857	0.857	1.000
Specificity	0.941	0.971	1.000	0.971	0.912	0.971

## 2 Experimental design, materials and methods

### 2.1 Samples of coffee and selections

The 25 selections differed in the proportion of specific defects and healthy coffee beans. Coffee quality specialists, bean by bean, manually picked out the whole and healthy beans, broken, sour and black beans, woods and skin of each selection (Fig. 2 presents photographs of each group). The counting was performed based on the weight of each previously mentioned group, in percentage (Table 1). Then, the samples were built by blending a portion of each selection (20% or 40%) with each bases (100% of Arabica (i), and two mixtures of Arabica to Robusta coffees in the proportions 80:20 (ii) and 50:50 (w/w) (iii)). Robusta 100% and the bases were also evaluated (Table 2). The final samples were roasted in a medium level of roasting (Probat Emmerich am Rhein, Germany, model PRG1Z, ERD Gas), until reaches 17% of weight loss and a luminosity, L*, between 22 and 26 (Konica Minolta portable colorimeter BC-10). After blending and roasting, the samples were ground (Ditting grinder KR805; Bachenbülach, Switzerland) on level 2.

### 2.2 FTIR-PAS analysis

The FTIR-PAS assessments were performed in a circular metal PAS cell of 9 mm diameter and 5 mm in depth containing the sample of RG coffee isolated from the room atmosphere with helium purging for 1 min before the analysis, reducing the water vapor and carbon dioxide in the sample chamber. After infrared light had been focused on sample, an ultrasensitive microphone detected the PAS signal. The resulted PAS spectrum was an average of 16 scans, with 4 cm^−1^ resolution in a wavenumber region of 600–4000 cm^−1^. Before analysis, the PAS signal was calibrated with a polyethylene standard sample. The PAS signal normalization process to a black body coated reference sample, commonly named as carbon-black, was performed for eliminating the influence of the non-uniform intensity of the light source spectrum. Thus, the FTIR-PAS normalized signal was the ratio between the sample PAS signal amplitude and the carbon-black PAS signal amplitude. The resulting signal, which generates the PAS spectrum, is dependent on the composition of the sample because it is directly proportional to the amount of light energy absorbed by the sample at each wavenumber.

### 2.3 Multivariate data analysis

PLS-DA (Partial Least Squares Regression - Discriminant Analysis) was applied for the set of PAS spectra of coffee samples. The optimum PLS-DA model dimensions were determined by the minimum RMSECV value for the calibration samples, obtained by the leave-one-out procedure with 108 samples. This procedure resulted in the choice of six latent variables for mean-centered model development. Table 3 presents specific parameters of this data analysis.
