# Supplementary material for: Data on roasted coffee with specific defects analyzed by infrared-photoacoustic spectroscopy and chemometrics
Source: Data Brief. 2018 Aug 9;20:242–9. doi: 10.1016/j.dib.2018.08.013 (PMC6104563; doi:10.1016/j.dib.2018.08.013)
Supplement: Supplementary file 2 — Supplementary material [file mmc2.docx]

Dear Editor,

We would like to bring to your notice that there are no interests to declare.

Rafael Carlos Eloy Dias, Patrícia Valderrama, Paulo Henrique Março, Maria Brigida dos Santos Scholz, Michael Edelmann, Chahan Yeretzian.
